# Intraocular Pressure Induced Retinal Changes Identified Using Synchrotron Infrared Microscopy

**DOI:** 10.1371/journal.pone.0164035

**Published:** 2016-10-06

**Authors:** Hsin-Hui Shen, Guei-Sheung Liu, Seong Hoong Chow, Jiang-Hui Wang, Zheng He, Christine Nguyen, Tsung-Wu Lin, Bang V. Bui

**Affiliations:** 1 Department of Materials Science and Engineering, Faculty of Engineering, Monash University, Clayton, Victoria, Australia; 2 Infection and Immunity Program, Biomedicine Discovery Institute and Department of Biochemistry and Molecular Biology, Monash University, Clayton, Victoria, Australia; 3 Centre for Eye Research Australia, Royal Victorian Eye and Ear Hospital, East Melbourne, Victoria, Australia; 4 Ophthalmology, Department of Surgery, University of Melbourne, East Melbourne, Victoria, Australia; 5 Department of Optometry & Vision Sciences, University of Melbourne, Parkville, Victoria, Australia; 6 Department of Chemistry, Tunghai University, Taichung City, Taiwan; University of Florida, UNITED STATES

## Abstract

Infrared (IR) spectroscopy has been used to quantify chemical and structural characteristics of a wide range of materials including biological tissues. In this study, we examined spatial changes in the chemical characteristics of rat retina in response to intraocular pressure (IOP) elevation using synchrotron infrared microscopy (SIRM), a non-destructive imaging approach. IOP elevation was induced by placing a suture around the eye of anaesthetised rats. Retinal sections were collected onto transparent CaF_2_ slides 10 days following IOP elevation. Using combined SIRM spectra and chemical mapping approaches it was possible to quantify IOP induced changes in protein conformation and chemical distribution in various layers of the rat retina. We showed that 10 days following IOP elevation there was an increase in lipid and protein levels in the inner nuclear layer (INL) and ganglion cell layer (GCL). IOP elevation also resulted in an increase in nucleic acids in the INL. Analysis of SIRM spectra revealed a shift in amide peaks to lower vibrational frequencies with a more prominent second shoulder, which is consistent with the presence of cell death in specific layers of the retina. These changes were more substantial in the INL and GCL layers compared with those occurring in the outer nuclear layer. These outcomes demonstrate the utility of SIRM to quantify the effect of IOP elevation on specific layers of the retina. Thus SIRM may be a useful tool for the study of localised tissue changes in glaucoma and other eye diseases.

## Introduction

Glaucoma is a relatively common age-related retinal neurodegenerative disease, estimated to affect over 80-million people worldwide [1]. It is characterised by the death of retinal ganglion cells, which convey visual information to the brain. Elevated intraocular pressure (IOP) and advancing age are robust risk factors for glaucomatous optic nerve damage [2]. Our understanding of the mechanisms underlying IOP-induced injury remains incomplete. However it is clear that the site of injury is at the optic nerve head and the layer most affected is the retinal ganglion cell layer. Thus there is a need for sensitive tools with the capacity to study localised changes such as those occurring in the ganglion cell layer. Such approaches have the potential to provide a deeper understanding of molecular and biochemical changes occurring in the retina with IOP elevation.

Non-invasive infrared (IR) spectroscopy has been used to quantify chemical and structural changes in a wide range of materials by detecting absorption of light across a wide spectrum of wavelengths (wavenumber ranging from 4,000 to 400 cm^-1^). IR spectroscopy coupled to a microscope (IR microscopy) is a powerful analytical technique that allows a two dimensional chemical map of an object to be developed without the need for any contrast agent or fluorescent probe. The addition of a synchrotron radiation source enhances brightness (300 x higher than a conventional IR source). The increased sensitivity associated with synchrotron IR spectromicroscopy (SIRM) has helped to provide novel insights into the pathogenesis of cancer [3], bone disease [4] and neurodegenerative disease of the brain [5].

Recently SIRM has been employed in eye research to study the differentiation of the various layers of the corneal epithelium [6–9]. SIRM imaging is well suited for analysis of the exquisitely layered structure of the retina. The chemical microstructure of retinal layers was first studied using SIRM by Wetzel *et al*. [10], which highlighted advantages associated with *in situ* chemical mapping (spatial map of individual chemical peaks) of retina layers. This approach has since been used to quantify changes in saturated and unsaturated fatty acids in neuronal layers of retinae from a murine model of Alzheimer’s disease [11, 12]. In order to obtain robust results, the retinal tissue in that study had to be pre-treated with reagents that had the potential to change the tissue and thus introduce contaminants. Mattson *et al*. [13] was able to avoid such confounds by imaging freshly thawed retinal sections. These authors showed that there was a robust nucleic acid peak at 1712 cm^-1^, but did not provide information about the wider spectrum, which can return information as to changes in lipids and proteins.

The very earliest changes to retinal ganglion cells associated with IOP elevation involve synaptic and dendritic changes [14–16], which at present are difficult to detect. Such subtle changes may manifest as localised modification of lipid, proteins and nucleic acids. In this study, we addressed this question by using SIRM to image freshly thawed rat retina collected 10 days following exposure to a short period of mildly elevated IOP. By analysing specific peaks in the SIRM spectra high-resolution SIRM maps of retinal sections can be derived. Such maps allowed us to examine how the biodistribution of molecules of lipids, proteins and nucleic acids is altered by IOP elevation.

## Materials and Methods

### Ethics approval and animal maintenance

All experimental procedures were conducted in accordance with the ARVO Statement for the Use of Animals in Ophthalmic and Vision Research and the Australian National Health and Medical Research Council Code of Practice for the Care and Use of Animals for Scientific Purposes. Animal ethics approval was obtained from the Animal Ethics Committee at The University of Melbourne (Ethics number: 13-044-UM).

Adult Long-Evans rats were bred and maintained at the rat facility of the Melbourne Brain Centre (Parkville, Victoria, Australia). Male rats were housed in a standard 12-h light (<40 lux)/ 12-h dark environment and had access to food and water *ad libitum*. All surgical and assessment procedures were undertaken in anaesthetised animals (intraperitoneal injection ketamine/xylazine, 55:5 mg/kg). During procedures body temperature was monitored and maintained at ~37°C.

### Experimental IOP elevation

**Fig 1:**
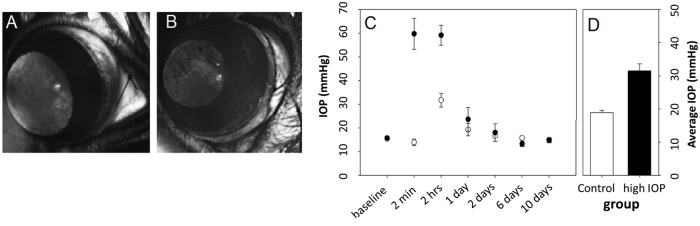
Induction of transient IOP elevation using a circumlimbal suture. **A:** In control eyes, the sham procedure involved loosely tying a suture around the eye behind the limbus. **B:** In treated (high IOP) eyes the suture was tied firmly behind the limbus to elevate IOP. **C:** IOP (mean ± SEM) was monitored for 10 days after suture application in high IOP (solid) and control (open) eyes (n = 6 each). **D:** Average IOP over the ten days was higher in treated compared with control eyes.

To demonstrate that SIRM is able to detect IOP-induced changes in chemical composition, imaging was undertaken of retinal tissue collected 10 days after treatment [17]. In anaesthetised rats one randomly selected eye underwent IOP elevation by securing a suture around the eye. The contralateral eye served as control. As shown in Fig 1A and 1B, a circumlimbal suture (8/0, nylon) was tied around the equator of the eye at a distance of approximately 1.5 mm behind the limbus. The suture was secured on the ocular surface via 5–6 subconjunctival anchor points. These anchor points were achieved by threading the suture under the crossing of major drainage veins that can be seen outside the eye. Care was taken to avoid the vortex veins. In the high IOP eyes, the suture was tightened firmly. In the control eyes, a sham procedure was performed, wherein the suture was loosely tied. Significant IOP elevation was produced at 2 minutes and at 2 hours after the suture application (Fig 1C). The average of all data points from 2 minutes after IOP elevation is shown in Fig 1D. The circumlimbal suture was left in place for 10 days after which retinae were harvested for SIRM imaging.

### Sample preparation

**Fig 2:**
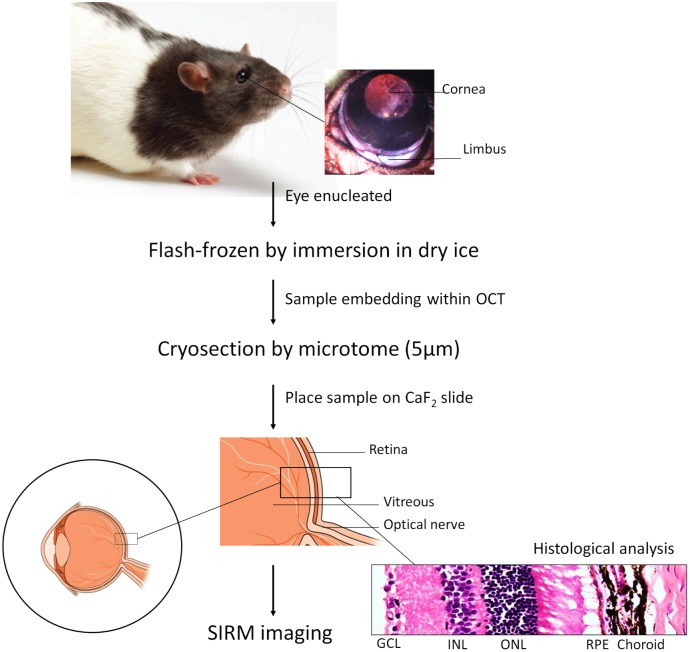
Preparation samples for SIRM imaging. Enucleated eyes were flash-frozen by immersion in dry ice and embedded for cryosectioning. 5 μm retinal sections were collected onto CaF_2_ slides for SIRM imaging.

The collection and preparation of retinal sections is schematised in Fig 2. Briefly, rats were euthanized by intraperitoneal injection of an overdose of sodium pentobarbital (Lethabarb, Virbac, Milperra, NSW, Australia). The eyes (12 eyes from 6 rats) were enucleated and flash-frozen in dry ice. Eyeballs were embedded within Optimal Cutting Temperature compound (Tissue-Tek OCT, Sakura Finetek, Torrance, CA, USA) embedding media and serial 5 μm thick sections were cut on a cryostat (Leica Microsystems, North Ryde, NSW, Australia) Sections that contained the optic nerve were collected and retained on infrared-transparent CaF_2_ slides (Crystran Ltd, Poole, Dorset, UK) for imaging. The basic histological features of rat retina were determined by haematoxylin and eosin (H&E) staining before being subjected to the SIRM absorption spectra analysis. All imaging was performed on native retinal tissue without any further fixation or staining to ensure that SIRM signals were not contaminated.

### SIRM measurements of freshly isolated retinal tissue

The SIRM spectra of the retinal samples were acquired using the Infrared Microspectroscopy (IRM) beam line at the Australian Synchrotron (Clayton, VIC, Australia). Measurements were taken on a Bruker Vertex V80v FTIR spectrometer coupled to a Hyperion 2000 IR microscope controlled via Bruker Opus 7.0 software (Bruker Optic, Etlingen, Germany). To reduce the influence of water in the air, the chamber was connected to a purging system. An aperture of 10 μm x 10 μm was selected to acquire a single spectrum. SIRM spectra were acquired in transmission mode with a spectral resolution of 4 cm^-1^ across a spectral range of 4000 to 600 cm^-1^, with 64 scans per spectrum. The recorded spectra were first processed with rubber band baseline correction, which was then followed by normalization to the amide I peak using Bruker OPUS 7.2 software (Bruker Optics) as has been described [18].

## Results

### Effect of elevated IOP on retinal chemical distribution, determined using SIRM maps

**Fig 3:**
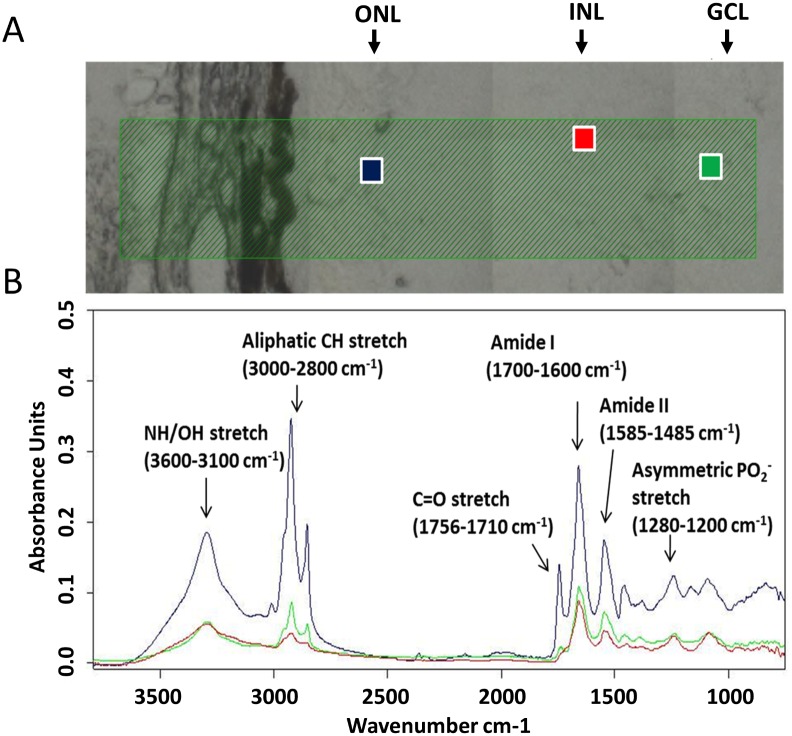
Selected regions of interests (A) and their relative SIRM spectra (B) in a normal retina. Spectra were recorded with an aperture of 10 μm x 10 μm. Blue: outer nuclear layer (ONL); Red: inner nuclear layer (INL); Green: ganglion cell layer (GCL). Spectra were normalized to the peak intensity of the amide I band.

Fig 3B shows SIRM absorption spectra for the regions of interest in the outer nuclear layer (ONL), inner nuclear layer (INL) and retinal ganglion cells layer (GCL) identified in Fig 3A. Molecular vibrations of chemical bonds showed distinct peaks at specific frequencies as per previous reports [18–22]. The broad band between 3600 and 3100 cm^-1^ corresponds to O-H and N-H stretching from water, proteins, and polysaccharides [23, 24]. The region between 3000 and 2800 cm^-1^ is known to originate from the symmetric and asymmetric stretching modes of methylene (-CH_2_-) and methyl groups (-CH_3_-) which are mainly made up of lipids [23]. The peak near 1737 cm^-1^ is due to ester carbonyl C = O stretching modes, which reflects the distribution of lipids. Between 1700 and 800 cm^-1^ the most prominent bands are amide I (1650 cm^-1^) and amide II (1541 cm^-1^), which originate from vibrations of the amide groups (-NH-CO-) found on proteins [18, 25]. The band at 1240 cm^-1^ is due to the phosphodiester groups (PO_2_) asymmetric stretching mode in nucleic acids [26]. Thus SIRM spectra could be clearly defined in freshly thawed retinal tissue to return distinct regional difference in the various layers of the retina. The spectra were consistent with typical spectrum as previously reported for retina [10, 11].

**Fig 4:**
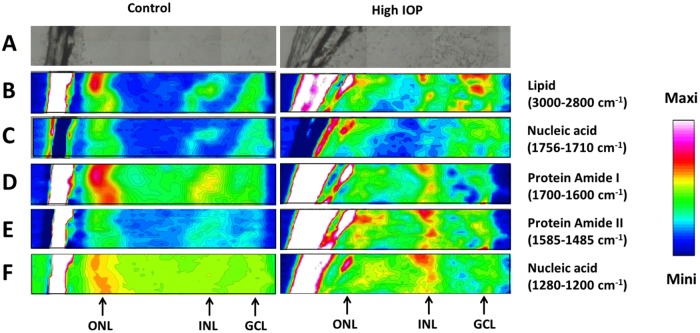
Normalized retinal SIRM maps from a control (left) and high IOP (right) eye. **A:** Position of analyzed area; **B:** Lipid (3000–2800 cm^-1^); **C:** Lipid (1756–1710 cm^-1^); **D:** Protein amide I (1700 - 1600cm^-1^); **E:** Protein amide II (1585–1485 cm^-1^); **F:** Nucleic acid (1280–1200 cm^-1^). The colored bar indicates the relative intensity of the integrated band, normalized to the maximum intensity in the image.

There were distinct regional differences in SIRM spectra (regions of interest indicated in Fig 3A) in various layers of the retina. Shown for a slice of retina is the distribution of lipids, proteins and nucleic acids, determined by integrating key bands in the chemical spectra (Fig 4B–4F). This demonstrates that SIRM maps return robust signals that are specific for the major cellular layers in the retina (ONL, INL and GCL) without need for stains or other biomarkers. All layers showed high levels of nucleic acids. The ONL showed a higher intensity for lipids (Fig 4B and 4C), proteins (Fig 4D) and nucleic acids (Fig 4E) compared with the INL and GCL, consistent with the higher density of cells (photoreceptors and horizontal cells) found in the ONL. In particular, the dense photoreceptor outer segments are known to be rich in lipids. The INL (where bipolar, horizontal and amacrine cells reside) showed moderate levels of protein amide (Fig 4D and 4E, left panels), and can be differentiated from the GCL (also contains retinal nerve fibres and displaced amacrine cells), which showed moderate levels of protein amide and lipid (Fig 4B).

In comparison to the control eyes (Fig 4, left panels), changes in the major chemical moieties as defined by alterations to the relative intensity of the SIRM maps can be seen for eyes that had undergone IOP elevation (Fig 4, right panels). In particular, IOP elevation reduced protein amide I levels (Fig 4D right panel) and increased protein amide II levels (Fig 4E right panel) in the ONL. Interestingly, an increased lipid (Fig 4B right panel) and protein (Fig 4D and 4E right panels) in both the INL and GCL of high IOP eyes could be observed. In addition, there was an increased level of nucleic acid in the INL of high IOP eye, as shown in Fig 4C (right panel).

### Protein conformational changes induced by IOP elevation, defined using SIRM spectra

**Fig 5:**
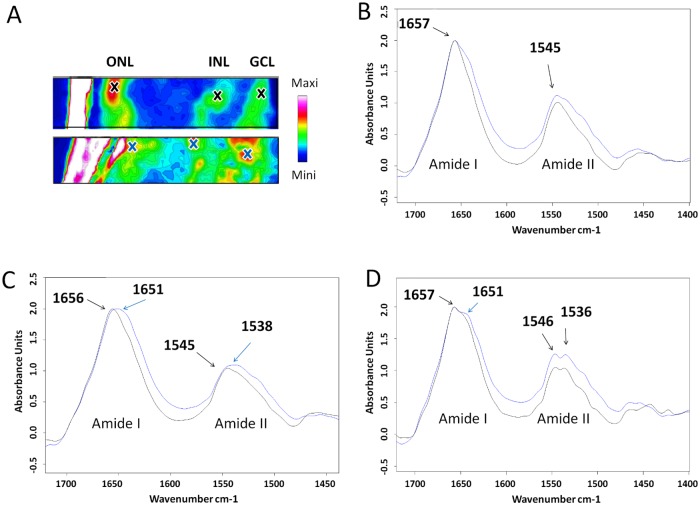
Changes to protein conformational in selected layers of the retina. **A:** Position of selected sites from control (A, top) and high IOP retinae (A, bottom) displayed on lipid maps. The colored bar indicates the relative intensity of the integrated bands. **B:** ONL protein spectra for control (black) and high IOP eyes (blue). Spectra were normalized according to the intensity of amide I band. **C:** INL protein spectra. **D:** GCL protein spectra.

The overall intensity of various SIRM spectra peaks can vary between eyes, whereas the shapes of SIRM spectra are more robust. Thus normalisation helps to reduce inter-sample variability. Fig 5A shows spectra from locations of peak intensity in the ONL, INL and GCL in a normal (top) and a high IOP (bottom) eye. Each spectrum has been normalized to the peak of the amide I band. Overall, the relative intensity of the amide II peak in IOP elevated retina (blue trace) was higher than the control eyes (black trace). Fig 5C (INL) and 5D (GCL) suggest that there was an extra shoulder around 1651 cm^-1^ in IOP elevated eyes indicating altered protein conformation [22]. There was a smaller difference in the overall spectra shape in the ONL between the control and high IOP eye (Fig 5B). Thus changes in protein conformation associated with IOP elevation appear to be greater in the inner retinal layers.

**Fig 6:**
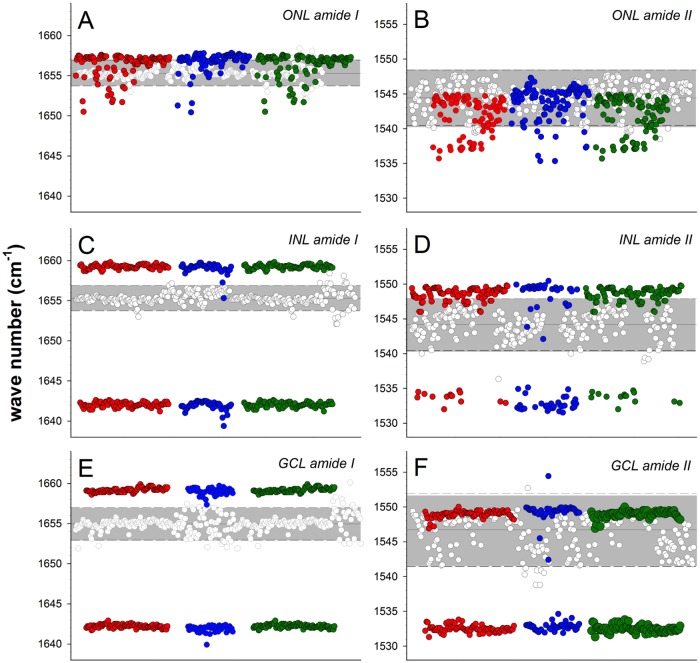
Effect of IOP elevation on amide I and amide II peaks in the ONL, INL and GCL. **A.** Amide I peaks in the ONL. Three different IOP treated eyes (each color represent one eye) are compared with all control eyes (white dots). The grey band indicates the 95% confidence limits for the control group. Data falling outside of these limits can be considered significant p < 0.05. **B.** Amide II peaks in the ONL. **C.** Amide I peaks in the INL. **D.** Amide II peaks in the INL. **E.** Amide I peaks in the GCL. **F.** Amide II peaks in the GCL.

To explore IOP induced alterations in protein structure across retinal layers, we examined amide I and II peaks in the ONL (Fig 6A and 6B), INL (Fig 6C and 6D) and GCL (Fig 6E and 6F) regions from four control and three high IOP eyes (Fig 5 and S1 Fig). In the ONL (Fig 6A and 6B), control eyes (white dots) showed amide I and II peaks at 1657 cm^-1^ and 1543 cm^-1^, respectively. IOP elevation produced a redistribution of amide I and amide II peaks to lower and higher vibrational frequencies, respectively. In the INL (Fig 6C and 6D) amide I and II peaks occurred at 1656 cm^-1^ and 1543 cm^-1^, respectively. Following IOP elevation two distinct amide I peaks at 1660 cm^-1^ and 1642 cm^-1^ could be identified in the INL (Fig 6C, each set of coloured dots represent one IOP treated eye). Similarly, two distinct amide II peaks were identifiable in the INL (Fig 6D) following IOP elevation.

In the GCL of control eyes, amide I peaks could be observed between 1650 cm^-1^ to 1658 cm^-1^ (Fig 6E), whereas amide II peaks were more variable ranging between 1538 cm^-1^ and 1553 cm^-1^ (Fig 6F). In high IOP treated eyes there was a distinct redistribution of the amide I peaks to higher and lower wavenumbers of 1658 cm^-1^ and 1642 cm^-1^ (colour dots), respectively. IOP elevation also appeared to have produced a redistribution of amide II into two distinct peaks, specifically at 1550 cm^-1^ and 1532 cm^-1^ (Fig 6F, colour dots).

**Fig 7:**
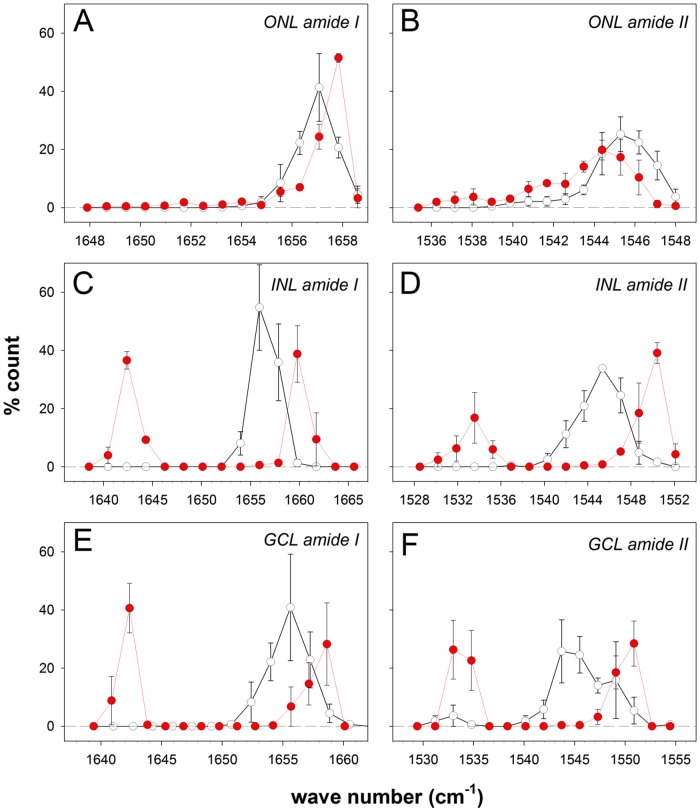
Effect on IOP elevation on the distribution of amide I and amide II peaks in retinal layers. **A.** Amide I peaks in the ONL for control (n = 4, whist symbols) and high IOP eyes (n = 3, red symbols) expressed as % (for ± standard error of the mean). **B.** Amide II peaks in the ONL. **C.** Amide I peaks in the INL. **D.** Amide II peaks in the INL. **E.** Amide I peaks in the GCL. **F.** Amide II peaks in the GCL.

Fig 7 summarises all the data points shown in Fig 6 into a frequency histogram to illustrate shifts in the peak wavenumber as well as the appearance of a second peak. In the ONL, in high IOP treated eyes 60% of locations analysed for amide I peak can be seen to have shifted from 1657 cm^-1^ to a higher wavenumber of ~1658 cm^-1^ (red dots, Fig 7A). In high IOP treated eyes there are a substantial number of locations in the ONL showing a shift in the amide II peak to lower wavenumbers (Fig 7B). Changes in the INL (Fig 7C and 7D) and GCL were more substantial (Fig 7E and 7F), where both amide I and II peaks showed a distinct second shoulder. This suggests the presence of conformational changes to proteins.

## Discussion

Our data show that SIRM imaging enables chemical mapping across freshly thawed retinal cross sections with enough spatial resolution to allow the key layers of the retina to be identified. Specifically, our chemical mapping approach revealed distinct biodistribution of lipids (peaks within the ranges of 3000–2800 cm^-1^ and 1756–1710 cm^-1^), proteins (1700–1600 cm^-1^ and 1585–1485 cm^-1^) and nucleic acids (1280–1200 cm^-1^) in specific retinal layers. Moreover, we showed that SIRM imaging is sensitive enough to quantify changes arising from a mild injury to the eye induced by IOP elevation, without need of exogenous probes. In particular, lipid, proteins and nucleic acid distributions changed within the inner retinal layers following a period of moderate IOP elevation. Of particular interest, the SIRM spectra revealed conformational changes to macromolecules (DNA, lipid and protein) that highlight the presence of cellular changes and perhaps cell death in the retina. These changes were more substantial in the inner (INL and GCL) compared with the outer retina (ONL) consistent with the injury induced by IOP elevation.

The inner retina and in particular the ganglion cells are known to be highly sensitive to IOP elevation [27]. The mechanisms of IOP-induced injury involve vascular and biomechanical stress [28, 29] acting on glia, connective tissue, blood vessel as well as neurons [30, 31]. IOP related stress is known to produce changes in cell and mitochondrial plasma membranes via excessive generation of Reactive Oxygen Species (ROS), including O^-2^, H_2_O_2_ and ·OH. Oxidative damage to macromolecules such as DNA, lipids [28, 29] and proteins [32] may underlie the changes to the SIRM spectra observed in our study. This is consistent with previous analysis of aqueous humor, lens, trabecular meshwork and retina collected from subjects with open angle glaucoma, which confirm the presence of increased lipid peroxidation [33, 34]. Consistent with these findings our results suggest that IOP elevation increased lipids in the INL and GCL. Along with these lipid changes we also detected increased protein in the INL and GCL, as well as increased nucleic acids in the INL of high IOP eye. Collectively, these inner retinal (INL and GCL) changes are consistent with previous data showing that the proximal retina is particularly sensitive to IOP elevation [27, 35, 36]. However, subtle IOP-induced changes were also seen in the outer retina, as evidenced by reduced protein amide I and increased protein amide II in the ONL. This would suggest that the injury generated by this model results in changes that are not entirely restricted to the inner layers of the retina [27].

Characterization of the early molecular events associated with retinal neurodegeneration has been one of the major aims of glaucoma research. The earliest cellular abnormalities are thought to involve synaptic changes [14] followed by progressive dendritic shrinkage, axon degeneration and finally loss of the cell body [37, 38]. The presence in the inner retina of an extra shoulder at both amide I (at 1642 cm^-1^) and amide II (at 1532 cm^-1^) peaks in addition to shifts in amide peaks to a lower vibrational frequency would suggest that IOP elevation resulted in a high degree of changes to protein conformation in the INL and GCL [22]. Shifts in the amide peak to a lower vibrational frequency along with the appearance of a shoulder have been shown to be indicative of cells undergoing apoptosis [22, 39–42]. An increase in the amide II peak relative to amide I (Fig 5) can arise from protein damage occurring during cellular differentiation or apoptosis [43].

In this study we assessed the effect of IOP elevation at 10 days after the initial surgical procedure. IOP was elevated at 2 minutes and 2 hours and had returned to normal within a day. This is a much less severe injury than had been reported previously. Liu *et al* (17) showed that 12 weeks of IOP elevation leads to ganglion cell loss, without any gross morphological changes to the retina [17]. Given that the length of injury employed here was substantially shorter than the previous report [17] the protein amide changes seen here are not likely to have been associated with gross morphological disruption to the retina. Consistent with this possibility, the sections shown in Fig 4A suggest that there was no gross disruption of retinal morphology. Previous reports [19, 39] suggest that changes to protein amide I and amide II peaks occur before gross morphological alterations. Whilst these subtle changes are consistent for the three IOP treated eyes, this limited sample size is a key limitation of this study. Moreover our analysis has been limited to one region of retina. It would be interesting to consider how changes in macromolecules can change as a function of retinal eccentricity, and time following IOP injury.

In conclusion, our preliminary study demonstrates the utility of SIRM for studies of IOP induced injury. Analyzing specific peaks in the spectra can return high-resolution SIRM maps indicative of macromolecules such as DNA, lipids and proteins to provide a better understanding of the response of specific retinal layers to injury. Thus SIRM may be a powerful tool for the study of localized tissue changes in eye diseases.

## Supporting Information

S1 Fig Normalized SIRM maps of retinal tissues from a representative control (left) and high IOP (right) eye. **A.** Position of analyzed area. **B.** Lipid (3000–2800 cm^-1^). **C.** Lipid (1756–1710 cm^-1^). **D.** Protein amide I (1700 - 1600cm^-1^). **E.** Protein amide II (1585–1485 cm^-1^); **F:** Nucleic acid (1280–1200 cm^-1^). The colored bar indicates the relative intensity of the integrated band, normalized to the maximum intensity in the image.
(TIFF)
