# Supplementary figures and images for: Intraocular Pressure Induced Retinal Changes Identified Using Synchrotron Infrared Microscopy
Source: PLoS One. 2016 Oct 6;11(10):e0164035. doi: 10.1371/journal.pone.0164035 (PMC5053542; doi:10.1371/journal.pone.0164035)

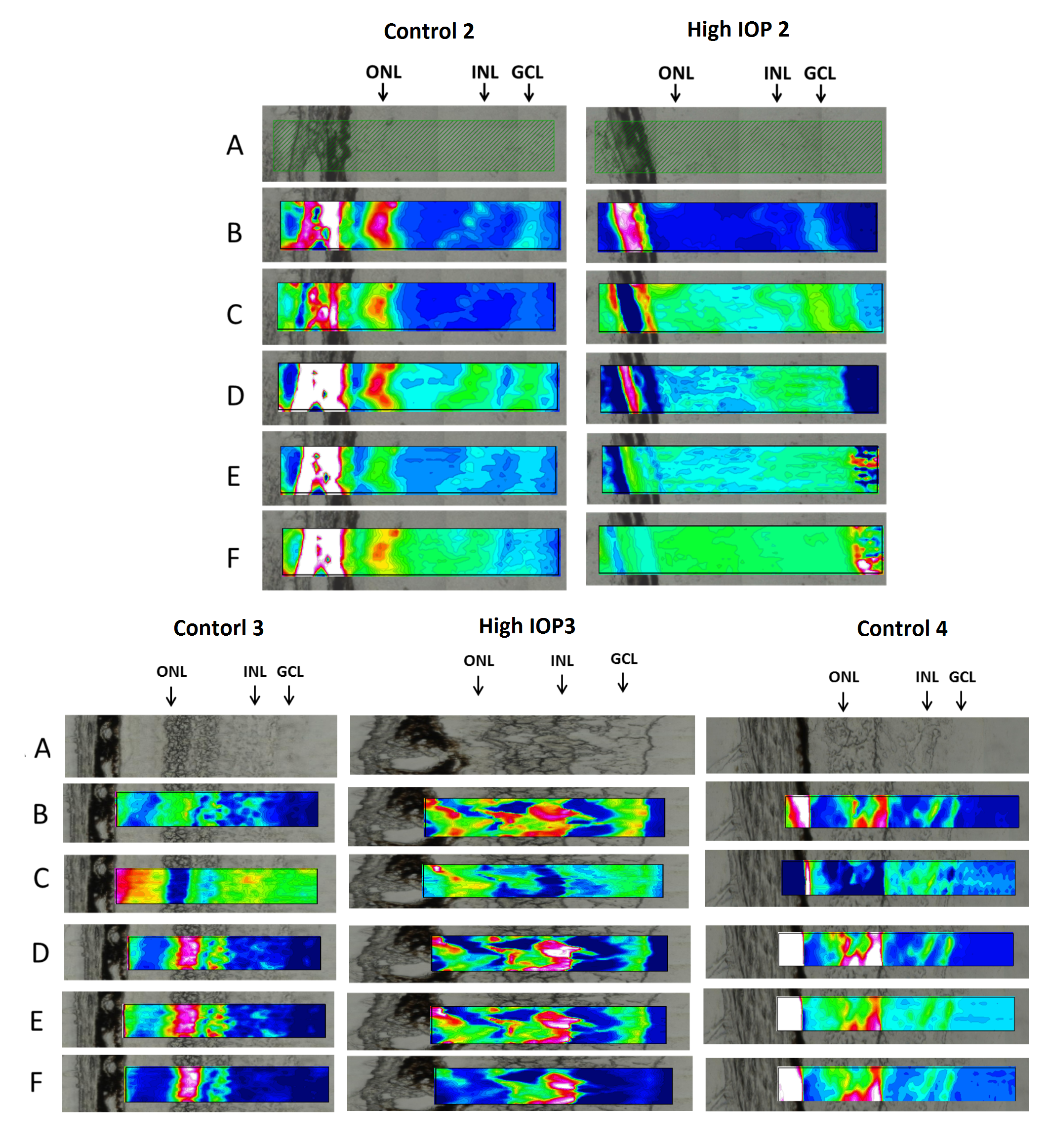

Supplement: S1 Fig — A. Position of analyzed area. B. Lipid (3000–2800 cm-1). C. Lipid (1756–1710 cm-1). D. Protein amide I (1700 - 1600cm-1). E. Protein amide II (1585–1485 cm-1); F: Nucleic acid (1280–1200 cm-1). The colored bar indicates the relative intensity of the integrated band, normalized to the maximum intensity in the image. (TIFF) [file pone.0164035.s001.tiff]
